# The economic burden of Borderline Personality Disorder: direct and indirect costs, and evidence from evaluations of interventions

**DOI:** 10.3389/fpsyt.2026.1835343

**Published:** 2026-09-30

**Authors:** Monica Ghoyal, Greta Dertwinkel, Katie Nicol, Ruchika Gajwani, Manuela Deidda

**Affiliations:** 1 School of Health and Wellbeing, University of Glasgow, Glasgow, United Kingdom; 2 Orygen, The National Centre of Excellence in Youth Mental Health, Melbourne, VIC, Australia; 3 Centre for Youth Mental Health, University of Melbourne, Melbourne, VIC, Australia; 4 National Health Service (NHS) Greater Glasgow and Clyde, Glasgow, United Kingdom; 5 Health Economics and Health Technology Assessment (HEHTA), Institute of Health and Wellbeing (IHW), University of Glasgow, Glasgow, United Kingdom

**Keywords:** Borderline Personality Disorder, BPD, direct and indirect cost, economic evaluations, health inequalities, healthcare costs

## Abstract

**Background:**

Borderline Personality Disorder (BPD) is characterized by longstanding patterns of difficulty in functioning, and when left untreated, BPD can impose a substantial financial burden on individuals and society, in terms of higher healthcare costs as well as broader societal costs.

**Aims:**

To conduct a critical review of the direct and indirect costs related to BPD. Additionally, to critically evaluate the economic evidence on interventions for BPD.

**Methods:**

A comprehensive search strategy was conducted across PsycINFO, PubMed, and SCOPUS. Studies were screened in two phases: an initial title and abstract review, followed by full-text screening against a predefined eligibility criteria.

**Results:**

Across 23 studies, high direct and indirect costs were consistently reported. Economic evaluations primarily examined established psychological interventions for BPD, such as Dialectical Behavior Therapy (DBT), Cognitive Behavior Therapy (CBT), and Schema-Focused Therapy (SFT), comparing costs and quality-of-life outcomes, with most demonstrating cost savings through reduced inpatient and emergency service use.

**Conclusions:**

This review highlights the substantial economic burden of BPD, driven by high service use, productivity losses, and unmet needs. While interventions such as DBT, CBT and SFT show potential cost-effectiveness, inconsistent cost definitions and the omission of key components, including informal care and long-term impairment, likely lead to an underestimation of true costs. More comprehensive and standardized economic evaluations are needed.

## 1 Introduction

Borderline Personality Disorder (BPD) is a serious mental illness characterized by persistent patterns of affective instability, an unstable self-image, fear of abandonment, impulsivity, and behavioral dysregulation (1). BPD is estimated to be present in 0.7–2.7% of adults over their lifetime, with higher rates of BPD observed in clinical settings, for example 22% among inpatient populations and 12% in outpatient services (2, 3). Onset typically peaks during adolescence and young adulthood (4), where symptoms can fluctuate over time, with periods of improvement and recurrence even without treatment (5). Individuals with BPD often experience complex unmet needs that contribute to long-term adverse effects, such as high-level functional impairment and premature mortality (6). Although research indicates that BPD diagnosis should not be controversial, it continues to be perceived as such by some, partly due to stigma and prejudicial attitudes toward the diagnosis (7). Additionally, terminology varies internationally; historically, ICD-based systems including those used in the United Kingdom and Europe, traditionally use Emotionally Unstable Personality Disorder (8), while more recent diagnostic frameworks, ICD-11 and the DSM-5 Alternative Model for Personality Disorders, adopt dimensional models (1, 9). Most economic studies rely on the categorical BPD construct, and this review uses the term BPD for consistency with the evidence base.

BPD poses a significant economic burden to the healthcare sector and society, in terms of direct and indirect costs (10). BPD is associated with substantial societal costs of €31,130 per person annually (11), reflecting both intensive service use and substantial productivity losses (12). These costs are substantially higher than those observed for other mental disorders (e.g. depression and alcohol dependence are associated with estimated costs of €7,139 and €1,110 annually per person respectively (13, 14)). People with BPD are disproportionately high service users, in comparison to those without BPD, with elevated rates of emergency presentations, repeated inpatient admissions, crisis-team involvement that concentrate spending in acute services (15). A ten-year prospective follow-up of the McLean Study of Adult Development cohort for those with BPD found sustained outpatient treatment use with more intermittent inpatient reliance, and although many individuals with BPD discontinued psychotherapy or medication at least once, most later resumed treatment, underscoring the fluctuating pattern of service use (16). In relation to indirect costs, individuals with BPD experience markedly disrupted work trajectories, spending a smaller proportion of adulthood in employment and facing far higher rates of job loss than their non-BPD peers, thereby driving significant productivity losses (17).

The considerable economic burden of BPD, together with its detrimental effects on quality of life (18, 19), emphasizes the urgent need for interventions that are not only effective in terms of leading to clinical benefits, such as improving functioning, reducing symptom severity and lowering self-harm and crisis use (20, 21), but also have good value for money, representing efficient use of limited healthcare resources. However, current economic evidence on BPD is limited.

Meuldijk et al. (22) conducted a systematic review of cost-evaluations for BPD interventions and results suggest that effective treatment is likely to be cost-effective, with potential reductions in healthcare and community service use. However, the review highlighted substantial methodological limitations, including heterogeneity in cost definitions and measurement, variation in study design and quality, and limited generalizability across healthcare systems, all of which restrict comparability and warrant caution in interpretation. Meuldijk et al. (22) noted that the number of economic evaluations remains small relative to the wider clinical literature on personality disorders. As their review included studies published only up to 2015, a critical review is needed as more recent psychological interventions, updated economic analyses, and advances in methodological approaches were not captured. Although previous literature highlights the large societal burden associated with BPD, key gaps remain in understanding the main direct and indirect cost drivers, and important components, such as informal care and criminal justice involvement are consistently excluded in cost calculations.

To our knowledge, no comprehensive critical review currently synthesizes these costs and as a result, important questions remain unresolved, including which factors account for the largest proportion of costs and whether key components are systematically captured. Overall, an up-to-date critical review is essential to critically synthesize literature on the main direct and indirect cost drivers for BPD and evaluate newer evidence on the cost-effectiveness of interventions for BPD. A critical review is particularly valuable in the context of BPD because the existing economic literature is fragmented, and methodologically heterogeneous in how costs and outcomes are measured and interpreted. Such an approach enables a deeper examination and critique of how economic evidence has been generated for BPD (23), it highlights gaps that have limited the field’s development, and clarifies where future research and resource allocation should be directed, functions that extend beyond the scope of a standard systematic review (24). Therefore, this critical review aims to critically evaluate: 1) the direct and indirect costs of BPD and 2) the economic evidence of interventions for BPD, including findings from full and partial economic evaluations.

## 2 Methods

This critical review adopted a systematic approach to identifying and gathering extant literature, which followed three stages: planning and searching, selection and data extraction, and evaluation. In line with the aims of this critical review, we explicitly defined our scope by focusing on studies involving individuals with BPD, examining direct and indirect costs, and evaluating economic evidence on interventions.

### 2.1 Planning and searching

Within the study team, specialists in health economics and mental health identified key themes to explore the aims of the research: to explore and critically evaluate 1) the costs of BPD, and 2) economic evaluation evidence of interventions for BPD. Guided by the study aims, key themes were translated into search strings, incorporating terms related to BPD and health economics. These were applied across relevant databases using predefined SIGN economic evaluation filters (25). The final search terms (**Table 1**) and the databases, capturing health economic and mental health research, were agreed upon between the study team and the university librarian. These included PsycINFO, PubMed and SCOPUS.

**Table 1:** Keywords used to search the databases.

Theme	Keywords
Clinical Focus (Borderline Personality Disorder)	‘Borderline Personality Disorder’, ‘BPD’, ‘BPD symptoms’, ‘BPD features’, ‘EUPD’, ‘Emotionally Unstable Personality Disorder’.
Health Economics (costs related to BPD and economic evaluations of interventions)	“Costs and cost analysis”, “Cost allocation”, “Cost control”, “Cost savings”, “Cost of illness”, “Cost sharing”, “Health care costs” “Direct service costs”, “Drug costs” “Employer health costs”, “Hospital costs”, “Health expenditures”, “Capital expenditures”, “Value of life”, “Economics, nursing”, “Economics, pharmaceutical”, “Fees and charges” “Socioeconomics”, “Cost benefit analysis”, “Cost effectiveness analysis” “Cost control”, “Economic aspect”, “Financial management”, “Health care cost”, “Health care financing”, “Health economics”, “Cost minimization analysis”, “Health resource allocation”, “Health resource utilization”, “Cost estimate”, “Cost variable” “Unit cost”, “Economics, hospital”, “Economics, medical”, “Economics”.

Search strings were adapted to align with the indexing structures of each database for the keywords targeted (see **Supplementary Table 1** for full search strings). After entering the search strings into each database, the searches yielded a total of 439 papers. After removing 129 duplicates, 310 papers were identified for further screening.

### 2.2 Selection and data extraction

In line with the eligibility criteria, studies were included in the critical review if they featured: individuals with BPD or features of BPD (including any age category), within any geographic location, indirect and direct costs of BPD and/or economic evaluations (partial and full) of interventions (including any mental health intervention for BPD and any comparison group), and if they were published in English or had an English translation. Due to the limited research available in this area, we did not apply an exclusion criterion based on the year of publication. Studies which examined several personality disorders, including BPD, were included, due to the lack of studies solely investigating BPD. Literature was excluded if: it did not relate to BPD and health economics, if it only mentioned one key concept (for example, only BPD without the association with health economics) and did not have any costs or rates of utilization (such as intervention costs, hospitalization or medication fees). Any articles which were not retrievable were excluded as the researchers were unable to screen the papers (see **Supplementary Table 2** for a summary of literature included in the review).

Literature identified from the initial search on the databases was uploaded and screened through Rayyan (46). Once duplicates were removed, articles were screened in two phases. First, the primary researcher (MG) manually screened titles, keywords and abstracts. This first phase focused on identifying the key themes in the articles, an aspect of costing or an economic evaluation of an intervention, and whether the eligibility criteria were met. For the second phase of screening after studies had satisfied the initial screening process, MG performed a full text screen to investigate the applicability of the articles to the critical review’s aims. Queries regarding inclusion/exclusion were discussed and agreed upon with the other authors.

### 2.3 Evaluation

Through searching the strings across three different databases, 439 articles were highlighted for screening. Papers were excluded if they did not meet the study’s inclusion criteria and if there were duplicates. After the first screening phase (abstract, title, and keyword screening against the inclusion criteria), 58 papers remained. Following the second phase of screening (full-text screening with the inclusion criteria applied) 23 papers remained (**Figure 1**). A data extraction table was employed to record detailed notes on the remaining 23 papers. This included the study design, aims, key themes, the indirect and direct costs of BPD, the costs of interventions, cost savings, and strengths and limitations of the study.

**Figure 1:**
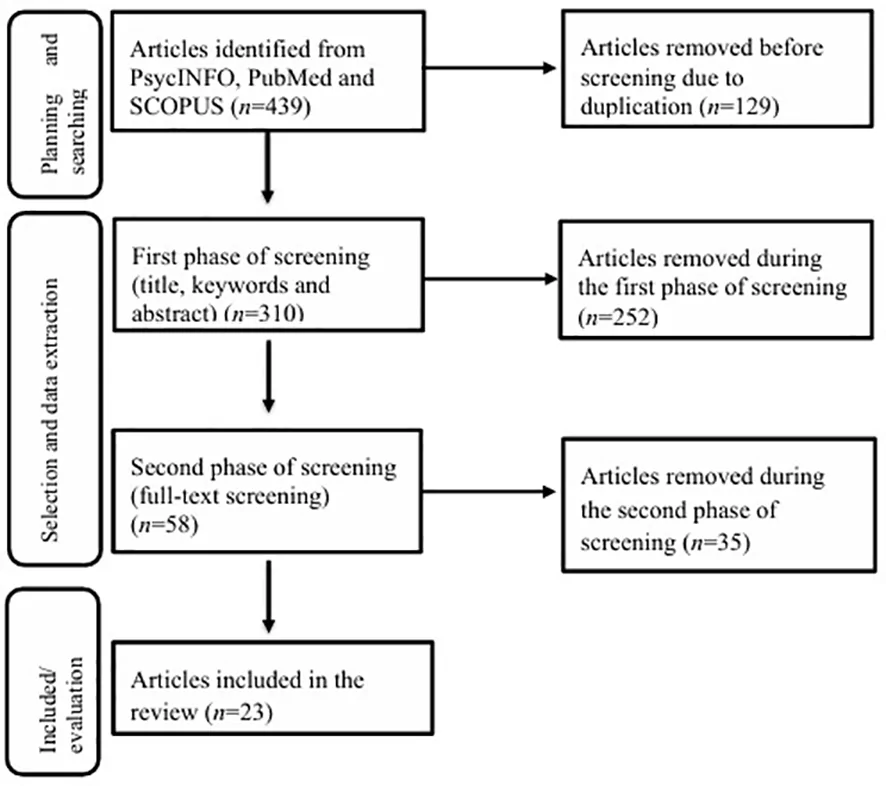
Figure 1 outlines the stages of the critical review process. A total of 439 records were identified through PsycINFO, PubMed, and SCOPUS. After removing 129 duplicates, 310 records proceeded to the first phase of screening (titles, keywords, and abstracts), during which 252 were excluded. 58 articles were assessed in the second phase of screening, with 35 removed. 23 articles met the inclusion criteria and were included for the critical review.

### 2.4 Patient and public engagement

Patients and members of the public were not involved in the design, analysis, or dissemination of this research and therefore consent was not obtained.

## 3 Results

Based on the 23 studies that remained after screening had taken place, 10 studies discussed the direct and/or indirect costs of BPD from a societal perspective, while 10 studies explored economic evaluations of different BPD treatments in comparison to treatment as usual (TAU) or costs pre-treatment, and three studies discussed both aims. Characteristics of the studies can be found in the **Supplementary Table 3**, which details additional information about the studies included in this review, including the design, geographic location of the studies and a summary of the key findings from all studies included in this critical review.

### 3.1 Costs associated with Borderline Personality Disorder

To answer the first research question, costs were considered specifically through: 1) direct costs associated with BPD within the healthcare system, and 2) indirect costs not directly associated with treatment for BPD.

Hastrup et al. (20) highlighted high total costs for individuals after a diagnosis of BPD in Denmark (€40,411), amounting to 16 times higher than individuals without a diagnosis (€2,440). Indirect costs amounted to 60% of the total for individuals with BPD (€23,184), specifically for public transfer costs, such as sick pay and unemployment benefits. For individuals with BPD, annual healthcare costs were €16,728, with approximately half attributable to mental health care; this was higher compared to those without BPD (€2,288). Compared to individuals without BPD, health care costs increased at the time of a diagnosis, compared to three years prior to a diagnosis and gradually declined over five years after a diagnosis. This surge in expenses was primarily due to psychiatric in-patient admissions and somatic care costs. Notably, psychiatric in-patient care accounted for half of all health care costs prior to a diagnosis of BPD, making it the most significant single cost category among individuals before a diagnosis of BPD. High combined direct and indirect costs were also highlighted by other literature (21, 34); for example, a German cost-of-illness study reported mean annual costs of €31,130 per person with BPD (11), with 49.2% attributable to medical costs, 5.5% to direct non-medical costs, and 45.3% (€14,086) to indirect costs. Costs for BPD exceeded those for Major Depressive Disorder (MDD). A German study reported mean annual costs of €4,636 vs €2,020 one year before diagnosis, €7,478 vs €3,638 one year after, and €11,817 vs €6,058 two years after diagnosis, respectively (28). To estimate the broader societal burden of BPD in a German population, a population-based cost-offset analysis (assuming a prevalence of 0.7%) projected total national costs of €8.88 billion, including €4.75 billion in direct costs and €3.93 billion in indirect costs (35).

#### 3.1.1 Direct costs

Studies report high healthcare costs for individuals with BPD compared to those without BPD. For example, in a Danish register-based cohort study, annual healthcare costs for individuals with early-onset BPD at age 20 were €12,574, compared to €1,475 for those without BPD (27). This relationship was evident in other studies, where similar figures were reported (11, 20, 29, 34). A prevalence-based micro study, assuming a 1% prevalence rate of BPD in the Irish population estimated that the annual total healthcare costs for treating BPD amounted to €311.5 million (26); however, this estimation presumes that all diagnosed individuals with BPD would use health services. Alongside high annual medical costs, those with BPD report high lifetime mental health service utilization; for example, the lifetime rate of receiving consultation or therapy in a community sample was 74.9% (43). Although service use is high, many with BPD may not be accessing health care consistently. For instance, in Tomko et al.’s (43) study the rate of service use for those with BPD for mental health care within an inpatient, emergency rehabilitation or crisis setting was approximately 15%, and for all mental health services was 3.2–34.9% in Bode et al.’s (28) study. Hence, total costs reported could be influenced by a smaller population of individuals accumulating disproportionately high costs.

Most direct healthcare costs were related to psychiatric care, where psychiatric inpatient costs were reported to be the highest, amounting to 75% of direct costs (11). Furthermore, Hastrup et al. (27) concluded that 55% of direct costs were related to psychiatric care, whereas Cailhol et al. (29) reported that 82.4% of direct costs were due to psychiatric care, including medication and hospitalization. Laurenssen et al. (34) identified 39.1% of direct costs as inpatient mental healthcare, with 18.6% for outpatient care and 14% for hospital day care. Bourke et al. (26) reported annual inpatient costs of €5,497 per individual with BPD, alongside high emergency department use (45% of individuals, averaging 2.3 visits in six months) and €3,282 in annual adult mental health service costs. In contrast, Bode et al. (28) found psychotherapeutic outpatient care to be the largest contributor, accounting for 69.3% of total direct healthcare costs. BPD, specifically in treatment seeking individuals, was the only personality disorder linked to high levels of health service costs, driven primarily by increased emergency service use before referral for treatment, which was significantly higher compared to other treatment-seeking individuals with personality disorders (32).

In a longitudinal study exploring chronic illnesses, health related lifestyle choices and service utilization, those with a diagnosis of BPD were significantly more likely to undergo medical hospitalizations, CT scans, X-rays and MRI scans compared to individuals with a previous diagnosis of BPD who no longer met clinical thresholds (42). Additionally, a higher percentage of participants with BPD reported having comorbidities, such as fibromyalgia, obesity, diabetes, chronic fatigue, osteoarthritis or multiple medical conditions in comparison to those who no longer met a BPD diagnosis. Furthermore, individuals with BPD reported increased medication use compared to those without a diagnosis (29); for example, Bourke et al. (26) indicated that 82% of individuals with BPD took one type of medication and 63% of individuals with a diagnosis of BPD were prescribed psychopharmacological medication for mental health difficulties (43). High healthcare costs were seen for spouses of individuals with BPD five years prior to a diagnosis, where the greatest expenditures associated with service utilization were in somatic and primary care, prescription medication, and psychiatric out- and in-patient care, which were significantly higher compared to spouses of individuals without BPD (20).

#### 3.1.2 Indirect costs

##### 3.1.2.1 Labor productivity, pensions and benefits

Higher unemployment rates in those with BPD were observed when compared with other mental health difficulties (28, 32). Hastrup et al. (27) illustrated that those with BPD were 22 times more likely to be unemployed, and 15 times more likely to receive a disability pension compared to those without a diagnosis (27). Annual productivity losses for individuals with BPD were estimated at €2,502.54 due to absence from work and €467.24 due to reduced efficiency (34). Wagner et al. (11) found that work disability represented the largest component of indirect costs, with 31.7% of individuals unable to work due to chronic somatic difficulties and BPD symptoms. Among those employed, 21.6% reported impaired work performance, averaging 75.3 sick days per year, equating to €6,882 annually. Disability pensions and unemployment benefits also contributed substantially to indirect costs, with many individuals transitioning from temporary payments (e.g. sick leave) to long-term disability or unemployment support following diagnosis (20). These patterns extended to spouses of individuals with BPD, who also incurred higher costs from disability pensions and unemployment benefits both before and after the individual’s diagnosis (20). It is relevant to highlight that most studies included in this review were conducted in countries with strong welfare systems (e.g., Denmark, Germany, the Netherlands), where access to state benefits such as disability pensions, sick leave, and unemployment support is available; consequently, indirect cost estimates partly reflect the structure of these welfare contexts, and such costs would differ in settings without comparable supports.

##### 3.1.2.2 Informal care costs

Spillover care costs can also be observed for close relatives of those with BPD, which can include health impacts on caregivers and informal care time costs (47). Spouses of individuals with BPD earned approximately two-thirds of the income reported by spouses of individuals without BPD (20). While spouses of those without BPD experienced average healthcare costs of €1,278 annually, comparable costs for spouses of individuals with BPD amounted to €18,287, of which 84% was attributed to lost productivity after the initial diagnosis of BPD. In Wagner et al.’s (11) study, 20.4% of participants with BPD in Germany received informal care from friends or family due to mental health difficulties, amounting to an average of 240.7 hours of care, costing €3,430 annually per individual.

##### 3.1.2.3 Criminality

Wagner et al. (11) reported that a year prior to outpatient treatment in Germany, 32.3% of individuals with BPD were involved in unlawful conduct. Of these incidents, 54.5% were related to destruction of property, 29.8% to traffic violations, and 15.6% to law enforcement activities, theft and physical harm; this amounted to an average cost of €381 per individual. However, there is a lack of health economic studies on BPD estimating the cost of crime and risky behavior. Hastrup et al. (20), for instance, did not include costs of criminality, which they highlighted as a serious limitation, underlining its implications on the reliability of BPD cost estimations. Furthermore, literature beyond this critical review illustrated high rates of BPD among prisoners, for example, the prevalence of BPD in prison populations was 15.6% (48).

### 3.2 Economic evaluations of interventions for Borderline Personality Disorder

To address the second aim, studies exploring the cost-effectiveness, cost-benefit and cost-of-illness of BPD interventions were examined. In this critical review, TAU typically consisted of clinical case management, assessment, community engagement, and psychiatric review, including inpatient psychiatric care (e.g., 44–46).

Across studies, Dialectical Behavior Therapy (DBT) was consistently associated with reduced healthcare costs and improved clinical outcomes for individuals with BPD (37). In an Irish cost‑effectiveness evaluation, incremental costs were compared with benefits to assess the cost‑effectiveness of DBT, measured through quality‑adjusted life years (QALYs) (33). QALYs are defined as the length of life weighted by quality of life and utility scores range from 1, indicating perfect health-related quality of life, to 0, representing death. Murphy et al. (33) found that DBT produced meaningful gains in health-related quality of life, with utility scores increasing from 0.49 at baseline to 0.69 at six-month follow-up. Although DBT was slightly more expensive than no-DBT over 18 months (€16,514 vs. €16,266), DBT generated an additional 0.2 QALYs and was considered cost-effective relative to national thresholds. Other studies also demonstrated substantial cost reductions following DBT for those with BPD, with employed participants showing reductions in work-related absenteeism (e.g., 21) and significant reductions in service utilization after DBT treatment, while being more cost and clinically effective than TAU (38). Similarly, Amner (30) conducted a cost-analysis evaluating service use costs of individuals with BPD a year before DBT, during DBT, and a year after intervention and found that 62% of individuals in the United Kingdom incurred lower or no costs after DBT, with hospital admission costs falling by 75% during treatment and by 20% post-treatment. Total costs decreased from £174,122 pre-treatment to £137,571 one year after DBT, with savings offsetting the DBT program. Amner (30) concluded that the DBT intervention was successful in reducing costs post-treatment for those with BPD.

Integrated models incorporating DBT also showed economic benefit. In Germany, Schindler et al. (41) compared Integrated Care BPD (ICB), which delivered DBT within assertive community treatment, with TAU, which resulted in lower annual healthcare costs than TAU (€18,369 vs. €23,759). Day-care inpatient costs were halved, and employment rates increased from 20% to 56% in the ICB group. Both groups showed improvements in psychosocial functioning and psychiatric symptoms, but ICB achieved these outcomes at lower cost. At the population level, a German cost-offset analysis estimated that DBT could generate annual savings of €305.33 million assuming 10% treatment uptake, with 55% of savings attributable to reductions in direct costs and 45% to indirect costs for those with BPD (35). DBT was estimated to reduce annual BPD costs per person to €8,860 compared to an estimated €16,230 for individuals with BPD who did not receive DBT.

While DBT remains the most extensively researched intervention for BPD, several other psychological treatments have also demonstrated cost savings (39) accompanied by reductions in service utilization. In the United Kingdom, psychoanalytically oriented partial hospitalization and TAU (general psychiatric care) significantly reduced annual costs (for the 18 months of care) compared to costs six months before treatment for those with BPD (31). The partial hospitalization group also showed notable savings from reduced medication use and emergency care (31). During the 18-month follow-up, annual costs remained substantially lower for the partial hospitalization group ($3,183 USD) compared with TAU ($15,490), with estimated savings of $12,000 per person relative to pre-treatment, effectively recouping program costs within two years. Additional evidence from a United Kingdom case study of long-term psychoanalytic psychotherapy also indicated substantial cost reductions, with annual healthcare costs decreasing from £6,769 before treatment to £1,569 after four years, alongside marked reductions in service use and cessation of emergency and police involvement (36). Hence, it was suggested that long-term psychoanalytic psychotherapy can reduce service utilization and costs throughout the treatment period and post-treatment.

Furthermore, a health economic evaluation based in the Netherlands compared mentalization-based treatment for those with BPD in a day hospital (MBT-DH) and an intensive outpatient (MBT-IOP) setting over 36 months (44). They reported that between baseline and 36 months, societal costs were €106,088 for MBT-DH and €91,368 for MBT-IOP. Although MBT-DH produced slightly more QALYs and significantly more months in remission, these benefits did not outweigh the additional costs. The authors concluded that MBT-DH was unlikely to be cost-effective relative to MBT-IOP. Finally, a cost-effectiveness analysis of outpatient treatments, Schema-focused therapy (SFT) and Transference-focused psychotherapy (TFP), were evaluated for those with BPD in the Netherlands over four years (45). Total per-person costs were lower for SFT (€37,826) than TFP (€46,795), while QALYs were broadly similar (2.15 vs. 2.27). SFT was therefore considered more cost-effective, although both treatments improved quality of life relative to baseline. Importantly, Van Asselt et al. (45) noted that most costs accrued outside the healthcare sector, such as social services, lost productivity, and out-of-pocket expenses, highlighting the value of a societal perspective in economic evaluations of BPD treatments.

The evidence for cognitive behavioural therapy (CBT) appeared to be more mixed. In a United Kingdom trial, Palmer et al. (38) found no statistically significant difference in total costs or QALYs between CBT plus TAU and TAU alone. Although CBT plus TAU was marginally less costly after adjusting for baseline differences (£689 lower) due to reduced inpatient admissions, it also produced slightly lower QALYs (–0.11) and therefore showed no clear cost-effectiveness advantage. A six-year follow-up of the same cohort reported high service use in both groups, with 54% of individuals with BPD requiring inpatient care and two-thirds of the individuals visiting emergency services, but noted shorter hospital stays in the CBT plus TAU group in comparison to TAU (10.81 vs. 60.97 days) (40). Reductions in suicidal behavior observed in the original trial persisted at follow-up, but overall QALYs remained lower in the CBT group, and CBT did not demonstrate cost-effectiveness, despite slightly lower long-term costs compared with TAU.

## 4 Discussion

This critical review aimed to explore and evaluate the costs of BPD and the cost-effectiveness and cost of interventions for BPD. Findings demonstrate that BPD is associated with a substantial and wide-ranging economic burden, with consistently elevated direct and indirect costs across healthcare, social care, and productivity domains. Secondly, there is evidence that the cost of intervention is likely to be offset by reductions in costs and service utilization, such as fewer emergency department presentations and reduced outpatient mental health service use. In this section, methodological weaknesses and gaps in the literature are discussed, alongside recommendations for future practice and implications.

### 4.1 Limitations in cost component selection and the consequences for economic estimates

A key methodological critique concerns the selection of factors included in estimates of direct costs. Several studies noted that variables underpinning total annual costs of BPD were often overlooked or excluded. For example, some studies only considered if participants had accessed their primary mental health care service, despite individuals often accessing additional health-related services not recorded (e.g., 19). Individuals with personality disorders were more likely to exhibit physical comorbidities, such as alcohol-related cardiomyopathy, and Cluster A and B disorders have been linked to self-reported physical health conditions including cardiovascular disease and arthritis (49). However, as studies on personality disorders, particularly BPD, are limited, healthcare use outside primary mental health remains poorly understood (49) and the presence of co-occurring physical health conditions can further obscure the attribution of additional costs (50).

In relation to indirect costs, individuals with BPD show frequent contact with criminal justice services (51), where prevalence rates are substantially elevated compared with the general population, and are higher among incarcerated women (27.4%) than men (18.8%) (48). However most economic evaluations did not include costs associated with criminality. Contact with the justice system not only generates immediate costs but also carries long-term consequences, such as reduced access to employment, housing, and social support (52). A difficulty in research for identifying these long-term costs could be related to challenges with attrition within BPD cohorts (53), as longitudinal studies could aid in identifying the indirect and long-term costs associated with BPD, such as crime and unemployment. However, these studies can be timelier and require more resources than shorter studies (54).

Another example of indirect cost factors not routinely included in research is productivity losses for both individuals with BPD and their family members (50). BPD literature highlights the significant impact of a BPD diagnosis on an individual’s family, particularly in relation to caring needs (55), yet these costs are not routinely included in economic evaluations. For instance, Hastrup et al. (20) only considered spouses, yet individuals with features of BPD may have more frequent but less stable relationships (56) and higher rates of divorce (57) compared to those without BPD. Consequently, costs in relation to interpersonal relationships, especially costs resulting from higher divorce rates, compensatory childcare or custody battles (58), as well as caring responsibilities (59, 60), are rarely captured (e.g., 21). Further research has predominantly focused on parents, especially mothers of those with BPD, where carers indicate a large burden in relation to financial difficulties, limitations on daily activities, and disruptions to work, family, and social life, suggesting significant lifestyle adjustments to meet caregiving demands (59). Carers also experience heightened subjective burden, grief, reduced empowerment, and increased rates of depression and anxiety (59), which can be harder to account for in economic calculations.

Taken together, these methodological limitations indicate that current economic evaluations capture only a narrow view of the true costs associated with BPD. Studies mainly focused on healthcare-related costs, omitting important indirect costs such as nursing home care, transportation, benefit payments (28), and criminality (32). The systematic omission of key cost domains, the challenges of measuring societal burden, and the heterogeneity of BPD presentations all contribute to a likely underestimation.

### 4.2 Eligibility criteria and study generalizability

The inclusion criteria applied in economic evaluations constitute a further methodological constraint, with many studies omitting individuals whose presentations are likely to generate significant costs. For example, some studies excluded “mildly” impacted individuals with BPD (20), or those with co-occurring mental disorders or symptoms, including major psychiatric disorders (11), dependency on drugs, or acute suicidality (21). Tate et al. (61) labeled psychiatric comorbidities as “a rule, rather than the exception” in BPD and empirical findings suggest that psychotic symptoms are common in BPD populations, with Pearse et al. (62) reporting that 80% of participants with BPD experienced psychotic symptoms. Furthermore, 95.7% of individuals with BPD have at least one additional psychiatric diagnosis (63), underscoring the complexity of accurately capturing costs. Some participants were only considered if they had met a diagnosis of BPD, or had been diagnosed (26), missing those who may have below threshold features of BPD. These sampling decisions can diverge from real-world service-use patterns; research in adolescents shows that subthreshold BPD presentations exhibit levels of psychopathological distress and health related quality of life impairment comparable to full-syndrome cases (64), and therefore are likely to contribute materially to healthcare utilization and costs. Such restrictive sampling limits the generalizability of findings, as the cost estimates produced could reflect only a narrow subset of the BPD population and therefore may carry substantial uncertainty, meaning the true cost of BPD is likely to be considerably higher than reported. It is important to note that some of the variation in cost estimates across economic studies could reflect the heterogeneity within BPD presentations (65); samples with higher levels of self-harm, comorbidity, or ICD-11 severe personality disorder features could be likely to incur markedly greater healthcare and societal costs than samples with lower-severity presentations, yet some studies treat BPD as a single, homogeneous category.

### 4.3 Functional impairment and accessing timely care

Functional outcomes are crucial, but they are often an under-captured component of the economic burden of BPD. Longstanding interpersonal and behavioral difficulties characteristic of the disorder substantially impair functioning and therefore generate indirect costs (19). Functional impairments in individuals with BPD can impact steady employment and the ability to sustain relationships (66). Although long-term cohort studies show high rates of symptomatic remission in BPD, functional recovery is far less consistent (67); for example a long-term prospective study of treatment seeking adults with BPD found that although symptoms commonly remitted over time, approximately 21% of participants achieved good levels of functioning after ten years (68). A meta-analysis of prospective studies examined long-term psychosocial functioning in adults with BPD and found moderate improvements in long-term functioning, although heterogeneity was high (5). The authors concluded that while symptoms tend to improve over time, gains in functioning are only slight in the long term. As most economic evaluations in this review focus on symptom reduction, with relatively few studies incorporating functioning outcomes, they risk overlooking downstream costs associated with the long-term negative impacts of BPD, such as disability, unemployment, productivity loss, and increased reliance on benefits and informal care. Early functional decline is also linked to poorer educational and occupational trajectories (27), which can compound lifetime costs. To better capture the full economic impact of BPD, future trials and cost-of-illness studies should routinely include validated measures of functioning and a longer follow-up period to quantify these indirect and long-term consequences.

It is evident that BPD is associated with significant costs, highlighting the urgent need for early and adequate treatment pathways. Research consistently demonstrates the effectiveness of psychotherapeutic treatments to reduce symptoms and produce cost offsets, yet their real-world impact is limited by long waiting lists, shortages of clinicians trained in therapies, geographic and commissioning inequities, and pervasive stigma that reduces help-seeking (69). These access barriers may prolong treatment for those with BPD, increase crisis contacts and inpatient use, and amplify productivity losses and reliance on social supports. Stigma further compounds these challenges, contributing to marginalization within healthcare, policy, and justice systems, and generating additional long-term costs.

### 4.4 Limitations, future directions and implications

In the interpretation of any health economic evaluation, context must be taken into consideration. The cost of BPD is likely to vary across geographic locations, influenced by welfare and labor market factors, as well as characteristics of the healthcare system, such as the average length of psychiatric stays. Cost estimates may also differ because BPD encompasses considerable heterogeneity, particularly when understood dimensionally, where variations in severity and trait profiles can be associated with different levels of support required over time, which can limit meaningful comparison between studies. Research in this field often occurs in isolated national settings where most studies reviewed were conducted in Denmark, Germany, and the Netherlands, countries known for having strong welfare systems and the validity of these findings in countries with less comprehensive welfare provision must be carefully assessed. In addition, older studies included in this review may not reflect current treatment practices or service structures. Nevertheless, these studies were retained due to the scarcity of research in this area and their value in illustrating historical cost patterns.

A key priority for future economic research on BPD is improving the conceptual and methodological consistency of how core cost constructs are measured. For example in relation to productivity loss, both the human capital approach and the friction cost approach are commonly used, but their differing assumptions produce vastly different estimations (70). Literature would be enriched through qualitative research which could provide depth of understanding about indirect costs, the lived impact of stigma, and the complexities of the long-term financial consequences. Lastly, all studies in this review focused on adult populations, despite BPD often emerging in adolescence (71). This represents a critical gap in research, as understanding the cost of BPD at different developmental stages could inform costs at different time points and highlights opportunities for early cost savings. Such information would provide policymakers with a more accurate picture of the economic burden across the lifespan. As a critical review, this article synthesizes and interprets diverse sources without the procedural safeguards of a formal quality appraisal, which may introduce some selection limitations (72); however, this approach remains well-suited to offering an integrated and interpretive perspective on the topic.

The majority of studies in this review relied on cross-sectional designs, limiting causal inference and restricting conclusions about long-term trajectories of costs. To better understand the enduring effects of intervention on BPD symptoms and/or functioning, and the associated costs, future economic evaluations should adopt designs that allow these long-term and cross-sectoral impacts to be measured, recognizing the extensive costs associated with BPD across healthcare, social services, education, and justice systems.

By clarifying both indirect and direct costs of BPD, this review can provide knowledge of where costs might occur, for example, the high use of inpatient care, where there may be gaps in reporting costs, and potential costs savings from intervention. Similarly, the omission of indirect costs limits the ability of policymakers to design comprehensive responses to addressing the full societal burden of BPD. Ultimately, policymakers should prioritize intervention for those with BPD, not only to meet the needs of these individuals but to reduce the economic burden of BPD to society. By adopting a broader and inclusive approach to cost evaluations, future research can provide the clarity needed to guide equitable service provision and sustainable policy decisions.

## 5 Conclusions

This critical review explored the direct and indirect costs associated with BPD, and the cost-effectiveness of interventions. Evidence consistently highlights that BPD imposes a substantial financial burden on individuals, families, healthcare systems, and society. Although methodological limitations mean that true costs are likely underestimated, BPD is associated with higher expenditures compared to those without BPD. At the same time, economic evaluations suggest that overall, interventions for BPD are likely to be cost-effective and lead to cost savings in terms of fewer hospital admissions, shorter inpatient stays, and reduced emergency service use. Also, individuals with BPD face multiple difficulties accessing adequate support, driving long-term costs higher.

BPD therefore represents a major public health concern that demands timely, evidence-based intervention and deserves more attention from policymakers, researchers, and clinicians alike. Future research should adopt longitudinal designs, capture broader societal costs, and examine expenditure across developmental stages, particularly during adolescence, to yield the greatest long-term savings and to provide the correct support.

## Data Availability

The original contributions presented in the study are included in the article/**Supplementary Material**. Further inquiries can be directed to the corresponding author.
