# Supplementary material for: The economic burden of Borderline Personality Disorder: direct and indirect costs, and evidence from evaluations of interventions
Source: Front Psychiatry. 2026 Sep 30;17:1835343. doi: 10.3389/fpsyt.2026.1835343 (PMC13662043; doi:10.3389/fpsyt.2026.1835343)
Supplement: Supplementary file 1 [file Table1.pdf]

Supplementary Material

1 Supplementary Data

Table 1. Search terms used for database searches in PubMed, Scopus and PsycINFO.

Database	**Health economics**		**Clinical focus**
PubMed	"Costs and cost analysis"[Title/Abstract] OR "Cost allocation"[Title/Abstract] OR "Cost control"[Title/Abstract] OR "Cost savings"[Title/Abstract] OR "Cost of illness"[Title/Abstract] OR "Cost sharing"[Title/Abstract] OR "Health care costs"[Title/Abstract] OR "Direct service costs"[Title/Abstract] OR "Drug costs"[Title/Abstract] OR "Employer health costs"[Title/Abstract] OR "Hospital costs"[Title/Abstract] OR "Health expenditures"[Title/Abstract] OR "Capital expenditures"[Title/Abstract] OR "Value of life"[Title/Abstract] OR "Economics, nursing"[Title/Abstract] OR "Economics,	AND	"Borderline Personality Disorder"[Title/Abstract] OR "BPD"[Title/Abstract] OR "BPD symptoms"[Title/Abstract] OR "BPD features"[Title/Abstract] OR "EUPD"[Title/Abstract] OR "Emotionally Unstable Personality Disorder"[Title/Abstract])"

	pharmaceutical"[Title/Abstract] OR "Fees and charges"[Title/Abstract] OR "Socioeconomics"[Title/Abstract] OR "Cost benefit analysis"[Title/Abstract] OR "Cost effectiveness analysis"[Title/Abstract] OR "Economic aspect"[Title/Abstract] OR "Financial management"[Title/Abstract] OR "Health care cost"[Title/Abstract] OR "Health care financing"[Title/Abstract] OR "Health economics"[Title/Abstract] OR "Cost minimization analysis"[Title/Abstract] OR "Health resource allocation"[Title/Abstract] OR "Health resource utilization"[Title/Abstract] OR (cost AND estimate*[Title/Abstract]) OR (cost AND variable[Title/Abstract]) OR (unit AND cost*[Title/Abstract]) OR "Economics, Hospital"[MeSH Terms] OR "Economics, Medical"[MeSH Terms] OR "Economics"[MeSH Terms]		
PsycINFO	XB("Costs and cost analysis" OR "Cost allocation" OR "Cost control"	AND	XB("Borderline Personality Disorder" OR "BPD" OR

	OR "Cost savings" OR "Cost of illness" OR "Cost sharing" OR "Health care costs" OR "Direct service costs" OR "Drug costs" OR "Employer health costs" OR "Hospital costs" OR "Health expenditures" OR "Capital expenditures" OR "Value of life" OR "Economics, nursing" OR "Economics, pharmaceutical" OR "Fees and charges" OR "Socioeconomics" OR "Cost benefit analysis" OR "Cost effectiveness analysis" OR "Economic aspect" OR "Health care cost" OR "Financial management" OR "Health care financing" OR "Health economics" OR "Cost minimization analysis" OR "Health resource allocation" OR "Health resource utilization" OR"(cost N1 estimate*) OR "(cost N1 variable)" OR "(unit N1 cost*)" OR "Hospital Economics" OR "Medical Economics" OR "Economics")		"BPD symptoms" OR "BPD features" OR "EUPD" OR "Emotionally Unstable Personality Disorder")
Scopus	TITLE-ABS-KEY("Costs and cost analysis" OR "Cost allocation" OR "Cost control" OR "Cost savings" OR "Cost of illness" OR "Cost sharing" OR "Health care costs" OR "Direct service costs" OR "Drug costs" OR "Employer health costs" OR "Hospital costs" OR	AND	TITLE-ABS-KEY("Borderline Personality Disorder" OR "BPD" OR "BPD symptoms" OR "BPD features" OR "EUPD" OR

	"Health expenditures" OR "Capital expenditures" OR "Value of life" OR "Economics, nursing" OR "Economics, pharmaceutical" OR "Fees and charges" OR "Socioeconomics" OR "Cost benefit analysis" OR "Cost effectiveness analysis" OR "Economic aspect" OR "Health care cost" OR "Financial management" OR "Health care financing" OR "Health economics" OR "Cost minimization analysis" OR "Health resource allocation" OR "Health resource utilization" OR “(cost W/1 estimate*)” OR “(cost W/1 variable*)” OR “(unit W/1 cost*)” OR “economics, hospital ” OR “ economics, medical” OR “economics”)		"Emotionally Unstable Personality Disorder")

Note: Full search terms used for database searches were conducted on 08.07.2025 and updated on 04.12.2025.

Table 2. Summary of literature included in the critical review.

Author(s)	Design/ health economic method	Country and sample	Aim	Summary of main finding	Aim addressed in the review

Wagner et al., 2022 (11)	Cost-of-illness	Germany; N = 167; female n=132; male n=35	Investigated costs from a societal perspective of individuals with BPD seeking treatment.	Annual societal costs for treatment-seeking BPD patients were high (€31,130 per person), with 49.2% direct costs and 45.3% indirect costs, driven mainly by psychiatric inpatient care and productivity losses. Indirect costs were primarily composed of work disability, sick leave, and informal care.	1
Bourke et al., 2021 (26)	Prevalence-based costing study	Ireland; N= 196; female n= 158; male n=38	To examine the use of direct health care resources of BPD and corresponding costs.	Annual direct healthcare costs were €10,844 per person; range €5,228–€20,609, with inpatient care the main driver, and national costs were estimated at €311.5 million.	1
Hastrup et al., 2022 (27)	Register-based cohort Study	Denmark; N=177 BPD sample; N = 677 controls	To investigate vocational and educational outcome, and health care costs of early onset BPD.	Early-onset BPD was associated with poorer educational and employment outcomes. By age 20, participants with BPD showed lower academic performance, greater disability pension use, and healthcare costs more than eight times higher than controls.	1
Hastrup et al., 2019 (20)	Register-based cohort study	Denmark; N= 2,756 BPD sample; N= 11,024 controls	To investigate direct health care costs, indirect costs and societal costs before and after a BPD diagnosis.	Individuals with BPD had total costs of €40,441, over 16 times higher in comparison to controls. Costs were elevated for at least five years before diagnosis, and spouses also showed increased healthcare use and	1

				productivity losses in comparison to control spouses.	
Bode et al., 2017 (28)	Cost of illness; retrospective study of claims data	Germany; BPD sample N=6,599; MDD sample N=26,396	To examine the costs due to the treatment of BPD in relation to MDD.	Individuals with BPD had annual healthcare costs more than twice as high in comparison to MDD controls (€7,478 vs. €3,638 a year after index diagnosis). Psychiatric inpatient care and outpatient psychotherapy were the main cost drivers in comparison to controls.	1
Wagner et al., 2014 (21)	Retrospective study; Cost of illness	Germany; N=47; female n=43; male n=4	Investigated societal cost-of-illness in BPD for three one-year periods: pre-treatment of DBT, DBT treatment and follow-up.	Mean annual societal costs were €28,026 in the year before DBT, €18,758 during DBT, and €14,750 in the follow-up year, showing substantial cost reductions in comparison to the year before DBT, mainly due to decreased inpatient treatment costs.	1/2
Cailhol et al., 2016 (29)	Retrospective study using an insurance database	France; BPD sample n=36; PD sample n=38; controls n=165	To compare the utilization of physical health services by individuals with BPD,	Individuals with BPD had higher somatic healthcare use in comparison to controls, including more medication prescriptions, medical consultations and hospitalization	1

			other PDs and matched controls.	days. Annual healthcare costs averaged €12,761, with 17.6% attributable to somatic care. No significant differences were observed in comparison to other PDs.	
Amner, 2012 (30)	Cost analysis	United Kingdom; N=21; female n=17; male n=4	To produce a DBT cost analysis within United Kingdom treatment provision considering costs before, during and after treatment for those with BPD.	Individuals with BPD showed substantial cost reductions in comparison to the pre-treatment year, with £36,000 saved by the end of the post-treatment year, mainly due to decreased inpatient use and community nursing contacts.	2
Bateman & Fonagy, 2003 (31)	Cost-Benefit Analysis	United Kingdom; partial hospitalization n=22; psychiatric care n=19	To examine health service utilization costs for individuals with BPD in the six months before point of randomization, during the 18 months of the study, and over the course of the 18-month follow-up.	Specialist partial hospitalization was no more expensive than TAU and cost reductions in the follow-up period were shown in comparison to TAU, due to less inpatient and emergency-room use.	2
Sveen et al., 2024 (32)	Cost of illness; register-based cohort study	Norway; N=798; BPD sample n= 267	To explore contributions of PDs on the level of societal cost components (health service costs and productivity loss).	Individuals with BPD had higher health service costs in comparison to other PDs, and BPD, avoidant PD, and unspecified PD were each associated with greater productivity loss. No specific PD uniquely	1

				explained the overall high societal costs.	
Murphy et al., 2020 (33)	Cost-effectiveness analysis	Ireland; N=196; female n=158; male n=38	To investigate the cost-effectiveness of DBT for treating BPD (pre-treatment, during treatment and post-treatment).	Over 18 months, DBT was more expensive (€393) and more effective (0.20 QALYs) in comparison to routine care. The low incremental cost-effectiveness ratio indicated short-term cost-effectiveness.	2
Laurensse n et al., 2016 (34)	Retrospective study to calculate burden of disease; RCT	Netherlands; N=403; female n= 332; male n=71	To calculate the burden of disease of individuals with BPD eligible for MBT.	Individuals with BPD had a high burden of disease, with low quality of life (EQ-5D = 0.48) and high annual costs (€16,879), including €13,405 direct and €3,474 indirect costs.	1
Wunsch et al., 2014 (35)	Prevalence- based approach; Cost off-set estimation	Germany; sample= N/A	To calculate a cost-offset estimation for BPD treatment and the financial benefits of DBT.	Untreated BPD costs €16.23 thousand per person and €8.68 billion annually. DBT generated large projected savings, with direct and indirect cost reductions totaling approximately €305 million (10% uptake in treatment) to €2.75 billion (90% uptake).	1/2
Turri & Andreatta, 2014 (36)	Cost-offset analysis	United Kingdom; case study	To investigate if long-term psychoanalytic psychotherapy can offset costs of psychiatric care for those with BPD.	Long-term psychoanalytic psychotherapy reduced mental health service use and costs in comparison to the pre-treatment period, with	2

				annual savings of £5,000 in the post-intervention year.	
Pasieczny & Connor, 2011 (37)	Cost-benefit analysis	Australia; N=90; female n=84; male n=6	To examine the clinical and cost effectiveness of providing DBT over TAU for BPD	DBT led to significant reductions in self-injury, emergency department visits, psychiatric admissions, and bed-days, with lower average treatment costs than TAU after six months.	2
Palmer et al., 2006 (38)	Cost-benefit analysis	United Kingdom; N=106	To evaluate the cost-effectiveness of CBT plus TAU compared to treatment as usual alone for individuals with BPD.	CBT showed slightly lower costs (−£689) but also lower QALYs (−0.11) than TAU; differences were small and not statistically significant.	2
Stevenson & Mearers, 1999 (39)	Cost-benefit study	Australia; N=30; female n=19; male n=11	To conduct a preliminary cost benefit study of the effect of outpatient psychotherapy for those with BPD.	Hospital admission costs dropped from $684,346 in the year before treatment to $41,424 in the year after, a saving of $21,431 per person. With therapy costing $13,000 per person, the net saving was $8,431 per person in the first post-treatment year.	2
Davidson et al., 2010 (40). Follow up from Palmer et	RCT follow up study; Cost-benefit analysis	United Kingdom; N=76; female/ male = N/A.	To examine the 6-year outcome of individuals with BPD who were randomized to 1 year of CBT or TAU.	At 6-year follow-up, CBT showed lower hospitalization and service-use costs than TAU, although no statistically significant cost-effectiveness advantage was demonstrated.	2

al., 2006 (38)					
Schindler et al., 2024 (41)	RCT	Germany; N=100; females =75; males= 25	To examine if Integrated Care Borderline (ICB) is more effective than TAU for those with BPD.	ICB led to an 89% reduction in psychiatric hospital days (vs. 41% in TAU) and lower annual hospital costs (€5,546 vs. €10,726). Employment improved significantly only in the ICB group.	1/2
Keuroghlian et al., 2013 (42)	Follow up study of longitudinal data	United States of America; N=264; female n= 213; male n= 51	To investigate the long-term relationship of chronic medical illnesses, health-related lifestyle choices, and health services utilization in BPD.	People with a diagnosis of BPD had more chronic medical conditions, more unhealthy lifestyle behaviors, and higher medical service use (emergency room visits, hospitalizations, imaging) than people who no longer met criteria for BPD.	1
Tomko et al., 2014 (43)	Prevalence-based study	United States of America; N=34,481; BPD n= 1,030; non-BPD n=33,451	To determine rates of health care utilization for those with BPD.	People with BPD showed high psychiatric comorbidity, greater treatment utilization (including outpatient, inpatient, and emergency services), and significant impairment in functioning.	1
Blankers et al., 2023 (44)	Cost-effectiveness analysis and cost utility	Netherlands; N= 243; day hospital mentalization-based treatment (MBT-DH) n= 70; intensive	To report on the health economic evaluation of MBT-DH in comparison to MBT-IOP for individuals with BPD and a RCT	MBT-DH compared to MBT-IOP produced slightly higher QALYs and more remission months, but at higher societal costs (€106,038 vs. €91,368). MBT-DH was unlikely to	2

		outpatient mentalization-based treatment (MBT-IOP) n=77	evaluating the effectiveness at 36 months.	be cost-effective compared with MBT-IOP.	
van Asselt et al., 2008 (45)	Cost-effectiveness analysis	Netherlands; N= 86; Schema-focused therapy (SFT) n= 44; transference-focused psychotherapy (TFP) n=42	To evaluate the cost-effectiveness of SFT in comparison to TFP for individuals with BPD.	SFT had lower costs ($37,826 vs. $46,795) and higher recovery rates (52% vs. 29%) than TFP. Although QALYs were slightly lower, SFT appeared cost-effective compared with TFP.	2

Note: 1= first aim of paper: what is the cost of BPD; 2= economic evidence of interventions for BPD; ½ = both aims were addressed in the paper; RCT= randomized control trial; MDD = Major depressive disorder; DBT= Dialectal behavior therapy; TAU= Treatment as usual; CBT = Cognitive behavioral therapy; BPD = Borderline personality disorder; PD = Personality disorder.
